# First Complete Genome Sequence of the Skin-Improving *Lactobacillus curvatus* Strain FBA2, Isolated from Fermented Vegetables, Determined by PacBio Single-Molecule Real-Time Technology

**DOI:** 10.1128/genomeA.00884-16

**Published:** 2016-09-01

**Authors:** Kazuma Nakano, Akino Shiroma, Hinako Tamotsu, Shun Ohki, Makiko Shimoji, Noriko Ashimine, Misuzu Shinzato, Maiko Minami, Tetsuhiro Nakanishi, Kuniko Teruya, Kazuhito Satou, Chise Suzuki, Hiromi Kimoto-Nira, Miho Kobayashi, Koko Mizumachi, Reiji Aoki, Satoshi Miyata, Kazue Yamamoto, Yasuyuki Ohtake, Tomoko Eguchi-Ogawa, Naoko Moriya, Tatsuro Hagi, Masaru Nomura, Takashi Hirano

**Affiliations:** a Okinawa Institute of Advanced Sciences, Uruma, Okinawa, Japan; b Institute of Livestock and Grassland Science, National Agriculture and Food Research Organization (NARO), Tsukuba, Ibaraki, Japan; c Western Region Agricultural Research Center, NARO, Fukuyama, Hiroshima, Japan; d Technology Research and Development Laboratory, Asahi Group Foods, Ltd., Moriya, Ibaraki, Japan; e Research and Development Center Strategy Office, Asahi Group Holdings, Ltd., Moriya, Ibaraki, Japan; f Research and Development Headquarters, Asahi Group Foods, Ltd., Shibuya, Tokyo, Japan; g Office of Evaluation, Headquarter, NARO, Tsukuba, Ibaraki, Japan

## Abstract

The first complete genome sequence of *Lactobacillus curvatus* was determined by PacBio RS II. The single circular chromosome (1,848,756 bp, G+C content of 42.1%) of *L. curvatus* FBA2, isolated from fermented vegetables, contained low G+C regions (26.9% minimum) and 43 sets of >1,000-bp identical sequence pairs. No plasmids were detected.

## GENOME ANNOUNCEMENT

*Lactobacillus* is the largest and most diverse genus among the lactic acid bacteria (LAB) (1, 2). Their natural habitat ranges from fermented dairy, meat, and plant products to the oral cavity, intestinal, and vaginal tracts of humans and animals (1, 3, 4). The genotypic and phenotypic diversity of LAB was acquired by gain of functions through horizontal gene transfer (HGT) from other LAB and loss of dispensable ancestral functions (1, 5). Mobile genetic elements such as insertion sequences and phages play an important role in HGT (1, 6).

*Lactobacillus curvatus* is a LAB that is most commonly associated with fermented products (7, 8). *L. curvatus* FBA2 (FBA2) examined herein was isolated from radish and carrot pickled with rice bran and salt (9). FBA2 was selected from 200 strains of LAB as a strain that enhanced the expression level of type I collagen and hyaluronan in human dermal fibroblasts. FBA2 improved moisture and elasticity of the skin of hairless mice, which were fed a low protein diet, and increased collagen peptide uptake in rat intestine (9–12). Use of FBA2 in skin-improving products was patented, and some food products containing FBA2 are commercialized in Japan (12).

Genomic information is the key to clarifying potential functions of *L. curvatus*; however, its complete genome sequence is not publicly available yet (http://www.ncbi.nlm.nih.gov/genome/genomes/10762). One of the four scaffold-level sequences, *L. curvatus* JCM 1096, has been determined using Ion PGM in 72 contigs (BBBQ01000000) (total 1,814,792 bp; average G+C content of 41.8%) (2, 7, 8). Here, we report the first complete genome sequence of *L. curvatus*, strain FBA2, determined by the single-molecule real-time (SMRT) technology (13).

The genomic DNA was extracted at the early log phase and purified using a PowerClean DNA cleanup kit (MO BIO Laboratories, Carlsbad, CA), followed by 20-kb library construction for P6-C4 chemistry with shearing. Size selection was not performed. Eight SMRT cells (each 240-min movies) were sequenced using the PacBio RS II platform (Pacific Biosciences, Menlo Park, CA). *De novo* assembly was performed using the hierarchical genome assembly process (HGAP) version 2 (14). A single circular contig representing one chromosome (1,848,756 bp, G+C content of 42.1%, and 1,847× coverage) was obtained. No plasmids were detected by assembly or gel electrophoresis, indicating that FBA2 does not include plasmids. The genome contained 43 sets of >1,000-bp identical sequence pairs (3,118-bp maximum), including 22 insertion sequences, low G+C regions (2,000 bp, G+C content of 26.9% minimum), and five prophage sequences. Such regions are difficult to reconstruct by short-read sequencers with PCR amplification (6, 15). PacBio RS II produced reads with an average of 4,290 bp and a maximum of 43,302 bp with uniform coverage. Multi-kilobase reads and unbiased G+C coverage resolved >1,000-bp identical sequence pairs and low G+C regions, respectively.

The complete genome sequence of *L. curvatus* FBA2 will help to elucidate the skin-improving mechanism of and the diversity among *L. curvatus*.

### Accession number(s).

The complete genome sequence of *L. curvatus* FBA2 has been deposited in DDBJ/ENA/GenBank under accession number CP016028.
